# Hounsfield unit and gray-white matter ratio estimates in pediatric normal controls used in studies evaluating head computed tomography

**DOI:** 10.1016/j.ynirp.2026.100346

**Published:** 2026-04-30

**Authors:** Kie Honjo, Geraldine Goco, Eva Ta, Suzanne Laughlin, Anne-Marie Guerguerian

**Affiliations:** a Neuroscience and Mental Health Program, Research Institute, The Hospital for Sick Children, Toronto, ON, Canada; b Department of Critical Care Medicine, The Hospital for Sick Children, University of Toronto, Toronto, ON, Canada; c Division of Neuroradiology, Department of Diagnostic Imaging, The Hospital for Sick Children, Medical Imaging Department, University of Toronto, Toronto, ON, Canada

**Keywords:** Pediatric neuroimaging, Head CT, Hounsfield unit (HU), Gray-white matter ratio (GWR), Age

## Abstract

Head computed tomography (CT) plays a key diagnostic role in assessing anatomically visible cerebral injury, monitoring the development of edema and ischemia, and, in some diseases, assisting in the prediction of outcome. More specifically, after a cardiac arrest, head CT imaging will reveal the impact of global hypoxic-ischemic injury. In adults, some have shown that the gray-white matter ratio (GWR) calculated with Hounsfield units (HU) may be valuable for prognostication. Few studies have investigated their use in the pediatric population, and even fewer have considered age and maturation in their analyses.

The objective of this study was to evaluate pediatric normal controls and report their HU and GWR values for use in future studies. Non-contrast head CT scans of children without neurological diseases were used. HU values were measured using predefined regions of interest and the circular dots method. GWR was estimated with 12 different formulas. Analyses were stratified by age (<2 and ≥ 2 years).

We analyzed 42 scans. HU in the caudate nucleus, cortical gray matter, and brain parenchyma were lower in younger (n = 18) compared to older children ≥2 years (n = 24). No HU differences between the age groups were measured in the putamen, thalamus, and white matter. GWR calculated with basal ganglia (caudate nucleus + putamen) and cortical gray matter was lower in the younger group.

Head CT measurements of HU and GWR change with age in children under 2 years. This feature was most relevant in gray matter areas. Future studies should evaluate which GWR formulas are most effective in assessing the severity of global hypoxic ischemic encephalopathy after cardiac arrest in the pediatric population.

## Highlights

• There is a paucity of studies reporting CT head gray-white matter ratio estimates in pediatrics compared to the abundance of studies in adults following cardiac arrest.
• In this study, we report on Hounsfield Units and gray-white matter ratios of head CTs from a sample of pediatric normal controls.
• We contrast the results obtained by applying our methods and a wide variety of methods published by others.
• First, we found that Hounsfield Units measured in pediatric normal controls less than 2 years old have lower values in the cortical gray matter and caudate nucleus compared to those in older children.
• Second, gray-white matter ratios estimated in pediatric normal controls less than 2 years old show lower values when calculated with cortical gray matter and/or basal ganglia compared to those in older children.
• Age in children, most importantly when less than 2 years, is essential when developing reference standards for GWR measurements.

## 1 Introduction

Head computed tomography (CT) plays an important role in neurocritical care, including in the acute phase of post-cardiac arrest care. The American Heart Association (AHA) guideline for pediatric post-cardiac arrest care recommends considering completing a head CT to identify loss of gray-white matter differentiation as a sign of severe cerebral edema (Topjian et al., 2019). There is published evidence in adult patients demonstrating the value of changes in Hounsfield Units (HU) in brain regions for detecting ischemia or edema following cardiac arrest (Torbey et al., 2000). Some have published lower HU in gray matter regions in patients with poor outcomes in adults (Kenda et al., 2021; Torbey et al., 2000) and in children (Lee et al., 2022; Starling et al., 2015; Yang et al., 2019a, Yang et al., 2019b). Gray-white matter ratio (GWR) is defined as the ratio between HU calculated with any region of gray matter (GM) divided by white matter (WM). Some propose that it may be a simple neuroimaging biomarker for brain injury and a predictor of poor neurological outcome after cardiac arrest (Callaway et al., 2015; Torbey et al., 2000). Low GWR correlates with risk of mortality (Case et al., 2024; Coppler et al., 2022; Metter et al., 2011) and predicts poor neurological outcome in survivors (Choi et al., 2008; Gentsch et al., 2015; Jeon et al., 2017; Kenda et al., 2021; Lee et al., 2016a, Lee et al., 2016b; Scheel et al., 2013; Lang et al., 2024)

In a pediatric study, GWR in patients was significantly different from normal controls, and GWR was associated with a poor outcome (Lee et al., 2022). The lack of accepted standards for selecting regions of interest and the absence of readily available automated approaches for front-line clinicians have been suggested as barriers to adoption. GWR formulas vary across pediatric and adult studies. Some formulas perform better than others in predicting outcomes (Callaway et al., 2015; Na et al., 2018; Wang et al., 2020; Lee et al., 2016a). HU may be measured in a region of interest (ROI) drawn manually or through automation, or by using a circular dot (which we call ‘DOT’) placed over anatomical regions of interest. HU has been used to quantitatively analyze tissue density on head CT scans following traumatic brain injury in young children by our group (Guerguerian et al., 2015). We hypothesized that the method using segmented ROI would be more accurate as it covers the entire area of interest, though using the method with circular dots (DOT method moving forward) is simpler. If we were to demonstrate that our DOT method is adequate, it may facilitate its adoption in clinical settings. Moreover, published studies provide little information on how maturation during early development may alter GWR estimates. We hypothesized that changes in myelination would influence these estimates and that younger children would exhibit different GWR patterns in key anatomical regions than older children.

The purposes of this study were: 1) to analyze age-related changes in regional HU characteristics in head CT imaging of normal pediatric controls; 2) to analyze GWR estimated with different formulas published in adult and pediatric studies; and 3) to compare the HU generated with ROI and DOT methods. Publishing these values in pediatric normal controls would disseminate this rare normative information, which is valuable to groups developing approaches to quantify abnormalities.

## 2 Methods

### 2.1 Study samples

**Fig. 1:**
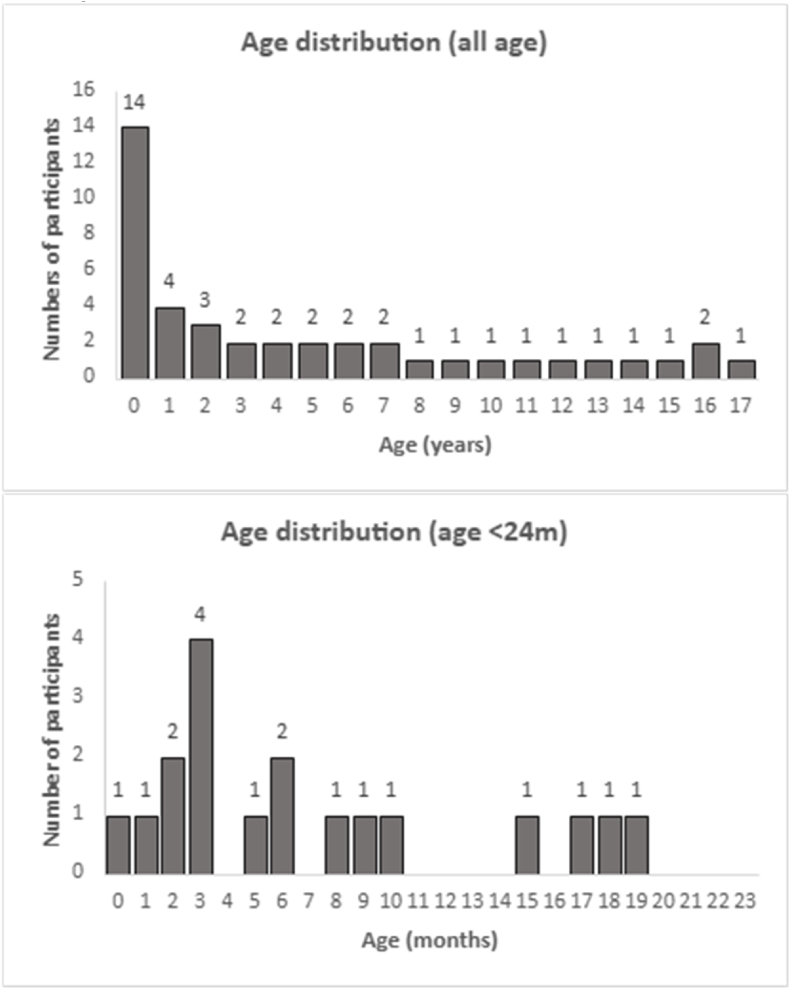
Normal cohort participants’ age distribution, in the top panel in years, and in the bottom panel in the first 2 years, in months. Frequency graphs illustrate the age distribution of participants and the number of participants in each age bin. Top panel: All participants between 0 and 18 years old (n = 42), breakdown by year of age. Bottom panel: Sample with age between 0 to 24 months (n = 18), breakdown by month of age.

Scanning healthy children with CT would not be approved for research in pediatrics because the potential risk of radiation exposure would be considered greater than minimal risk (Ethical Conduct of Clinical Research Involving Children 2004). To obtain a cohort of normal controls, we obtained ethics board approval to use previously acquired clinical imaging. We screened the institutional clinical radiology archive for CT head reports. Normal neuroimaging controls were selected from the clinical picture archiving and communication system (PACS) of the Hospital for Sick Children. We excluded patients with a positive CT scan or a neurologic diagnosis documented in medical records. These consisted of children (less than 18 years) who underwent head CT scans from 2003 to 2021 to exclude brain pathology as part of their clinical care. Final clinical reports were used to screen and select children with no known neurologic, neurocognitive, or developmental deficits and no visible abnormality on their CT scans. Following this selection, a board-certified experienced pediatric neuroradiologist (SL) and neurologist (KH) confirmed that all CTs were visually normal. Scans were excluded if there was any indication in the medical record of a head injury within the last 12 months before the index scan. We were unable to find scans across the entire age range (See age distribution in Fig. 1).

### 2.2 Head CT analysis

Head CT scans were acquired on clinical scanners (GE Healthcare, Lightspeed Ultra or Discovery CT750 HD, Milwaukee, Wisconsin) with age-appropriate head CT pediatric protocols (100 kVp or 120 kVp, a slice thickness of 5 mm). Non-contrast axial scans were visually inspected for the presence of any injuries, including intracranial hemorrhage, infarction, edema, ventricular changes, and bone trauma. Measurements of attenuation of the CT images were performed using the Medical Image Processing Analysis and Visualization (MIPAV) software version 11.0.8-2023-11-16 (McAuliffe et al., 2001). All CT scans were analyzed with the rater (KH) blinded to patients’ medical reports, medical histories, and clinical outcomes to reduce ascertainment bias.

**Fig. 2:**
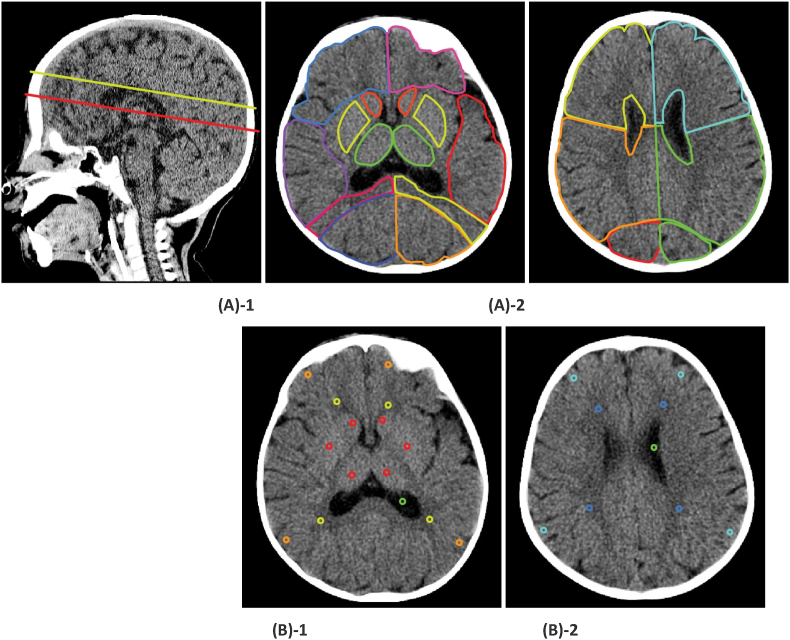
Examples of region of interest (ROI) and circular dot (DOT) methods. Sagittal scout image (top left) showing slice 1 (red line), where the 3rd ventricle and the basal ganglia (BG) were observed on axial scan ((A)-1, (B)-1) and slice 2 (yellow line), where the body of the bilateral ventricles can be first seen from the top of the brain on axial scan ((A)-2, (B)-2). (A)Examples of ROI in both slices. (B) Examples of DOT in both slices.

To proceed to the analysis, two slices are selected as follows: slice 1 (SL1), where the 3rd ventricle and the basal ganglia (BG) are visible; and slice 2 (SL2), where the body of bilateral ventricles can first be seen from the top of the brain (see Fig. 2) (Ta, 2015). We delineate ROI and circular dots and estimate HU within the areas; bilateral HUs from the same anatomical region are averaged for each slice. We draw ROI in the following sequence on each slice: first, we remove the bone and then delineate the whole brain parenchyma; second, we draw the ventricles; third, we draw each anatomical lobe and deep gray matter area's ROI. Regions are traced bilaterally in SL1 and include the caudate nucleus (CN), putamen (PT), thalamus (TM), frontal lobes, temporal lobes, parietal lobes, and occipital lobes. Regions traced in SL2 include the frontal lobes, parietal lobes, and occipital lobes. Finally, we draw round circular dots with a diameter of 2.4 to 2.8 mm that we call the DOT method. The DOT regions are placed bilaterally in SL1 as follows: CN, PT, TM, the frontal and parieto-occipital cortex (GM-SL1), frontal periventricular white matter (WM-fr1) and the posterior internal capsule (WM-SL1-posterior). The DOT regions are placed bilaterally in SL2 as follows: on the cortex (GM-SL2), and the deep WM in SL2 (WM-SL2) (see Fig. 2) (Ta, 2015).

### 2.3 Gray-white matter ratio (GWR) calculation

GWRs were calculated using the methods described above and other formulas reported in the literature. These are tabulated in Table 1. GWR published by others are calculated using one to three slices, including the following: 1) BG; 2) centrum semiovale; and 3) high convexity level. We carefully matched the GM and WM to the closest reported regions and adjusted each formula accordingly. GWR using different areas were the following: 1) GWR at BG (GWR_bg); 2) GWR with single nucleus at BG (GWR_simple); 3) GWR at cortical (GWR_cortical); and 4) GWR average of BG and cortical (GWR_average). In total, we calculated GWR with 12 different formulas as follows: 1) GWR_bg: GWR_bg 1 = (CN + PT)/WM-SL1, GWR_bg 2 = (CN + PT + TM)/WM-SL1; 2) GWR_simple: GWR_si (PT/PLIC) = PT/WM-SL1-posterior, GWR_si (PT/WM-fr1) = PT/WM-SL1-anterior, GWR_si (CN/PLIC) = CN/WM-SL1-posterior, GWR_si (CN/WM-fr1) = CN/WM-SL1-anterior, GWR_si (CN/WM-SL2) = CN/WM-SL2, GWR_si (PT/WM-SL2) = PT/WM-SL2, GWR_si (TM/WM-SL2) = TM/WM-SL2; 3) GWR_cortical = (GM-SL1 + GM-SL2)/(WM-SL1 + WM-SL2); 4) GWR_average: GWR + average1 = (CN + PT + GM-SL1 + GM-SL2)/(WM-SL1 + WM-SL2), GWR + average2 = (GWR_bg 1 + GWR_cortical)/2. Other formulas exist; most reported are similar to this dozen or employ automatic GM and WM segmentation methods. Table 1 also lists a fully automated approach that we could not test (Hanning et al., 2016). We adapted formulas by using only two slices when three slices were reported (Gentsch et al., 2015; Lee et al., 2016a, Lee et al., 2016b, 2022; Metter et al., 2011; Scheel et al., 2013; Wang et al., 2021; Yang et al., 2019a, Yang et al., 2019b), and by selecting deep WM at BG level (SL1 in our method). The selection of GM was identical to the previous studies except for one formula, i.e., GWR_cortical, as the original formula used cortical GM from the centrum semiovale level (similar to SL2 in our method) and a higher convexity level, which is not present in our method (Gentsch et al., 2015; Hrdlicka et al., 2023; Kawai et al., 2023; Kenda et al., 2021; Lee et al., 2016a, Lee et al., 2016b, 2022; Metter et al., 2011; Scheel et al., 2013; Wang et al., 2021; Yang et al., 2019a, Yang et al., 2019b). We adapted the formula by using cortical GM from SL1 and SL2.

**Table 1:** Formulas for calculating the Gray-white matter ratio (GWR).

GWR	Current study	Reported Formula	References
**GWR_bg 1**	(CN + PT)/WM-SL1	(CN + PT)/(CC + PLIC)	Lee et al., 2016a, Lee et al., 2016b; Gentsch et al. (2015); Scheel et al. (2013); Metter et al. (2011); Jeon et al. (2017); Wang et al. (2021); Kawai et al. (2023); Lee et al. (2022)∗; Yang et al. (2019b)∗; Starling et al. (2015)∗∗∗∗
**GWR_bg 2**	(CN + PT + TM)/WM-SL1	[(Pallidum + TH + CN + PT)/4] /[(PLIC + RLIC + ALIC) 3]	Kenda et al. (2021) ∗∗
**GWR_simple**	PT/WM-SL1-posterior	PT/PLIC	Lee et al., 2016a, Lee et al., 2016b; Gentsch et al. (2015); Scheel et al. (2013); Metter et al. (2011); Jeon et al. (2017); Kenda et al. (2021)∗∗; Hanning et al. (2016); Yang et al. (2019b)∗; Tetsuhara et al. (2021)∗∗∗∗
	PT/WM-SL1-anterior	PT/CC	Lee et al. (2016a); Jeon et al. (2017); Yang et al. (2019a)∗
	CN/WM-SL1-posterior	CN/PLIC	Lee et al. (2016a); Jeon et al. (2017); Choi et al. (2008)∗∗∗; Torbey et al. (2000); Coppler et al. (2022); Case et al. (2024); Yang et al. (2019a)∗
	CN/WM-SL1-anterior	CN/CC	Jeon et al. (2017); Yang et al. (2019a)∗
	CN, PT, TM/WM-SL2	CN, PT, TM/MWM1	Hrdlicka et al. (2023) ∗∗∗
**GWR_cortical (GWR_cerebrum)**	(GM-SL1+GM-SL2) /(WM-SL1+WM-SL2)	(MC1+MC2)/(MWM1+MWM2)	Lee et al., 2016a, Lee et al., 2016b; Gentsch et al. (2015); Scheel et al. (2013); Metter et al. (2011); Wang et al. (2021); Kawai et al. (2023); Kenda et al. (2021)∗∗; Hrdlicka et al. (2023) ∗∗∗; Lee et al. (2022)∗; Yang et al. (2019b)∗
**GWR_average 1**	(CN + PT + GM-SL1+GM-SL2)/(WM-SL1+WM-SL2)	(CN + PT + MC1+MC2)/(CC + PLIC + MWM1+MWM2)	Lee et al. (2016b)
**GWR_average 2**	(GWR_bg1+GWR_cortical)/2	(GWR_bg + GWR_cortical)/2	Lee et al. (2016a); Wang et al. (2021); Gentsch et al. (2015); Scheel et al. (2013); Metter et al. (2011); Kawai et al. (2023); Lee et al. (2022)∗; Yang et al. (2019b)∗
**GWR auto**	n/a	Whole brain mean GM/WM	Hanning et al. (2016)

CN, caudate nucleus; PT, putamen; CC, corpus callosum; PLIC, posterior limb of the internal capsule (IC); RLIC, Retrolenticular limb of IC; ALIC, anterior limb of IC; WM-SL1 White matter in slice 1; MC, medial cortex; MWM, medial white matter (MC and MWM have 2 levels: 1-level of centrum semiovale; 2- level of high convexity).

∗Pediatric studies; ∗∗ Kenda M and Hanning U used automatic region of interest mapping while others used manual method. Most studies use 3 slices, others used (∗∗∗) such as Hrdlicka J (2023) 2 slices and Choi SP (2008), Tetsuhara K (2021), and Starling RM (2016) used a single slice.

### 2.4 Data analysis

We averaged HU and GWR using medians and interquartile ranges (IQR). We evaluated the distribution and tested for normality. We stratified the sample into two age groups: young (<2 years) and older children (>2 years). Comparisons between age groups were tested; unpaired *t*-test or Mann-Whitney *U* test were used for normal or non-normally distributed data respectively; p values are reported with preset statistical significance p < 0.05 and Bonferroni correction was applied for multiple comparisons.

A Bland-Altman plot was created to compare the HU difference between our ROI and DOT methods in SL1 and SL2, in ventricles and three nuclei (CN, PT, TM). The x-axis of the plot is the HU measured by the ROI and DOT; the y-axis shows the difference between the average DOT measurement and the average ROI measurement. The mean difference between the average measurements for both methods was calculated to assess systematic bias. The upper and lower limits of agreement were plotted as 1.96 times the standard deviations from the mean difference of the two measurements. All scatterplots, box and whisker plots, and Blant-Altman plots were generated in Excel (Microsoft) for Windows 11, Version 16.0, and statistical analyses were performed with R, v.4.2.2 (R Foundation for Statistical Computing, Vienna, Austria).

## 3 Results

### 3.1 Demographics

A cohort of 42 head CT scans of normal children was identified. We show the age distribution (see Fig. 1): 14 subjects (33%) are less than 1 year old; 5 subjects (12%) are between 1 and 2 years old; and less than 3 subjects are included in each single year's bin between 2 and 17 years of age. Across the 42 subjects, the median age is 2.8 years (IQR 0.5-8.2). Among the younger group (<2 years) (n = 18), the median age is 0.46 years (IQR 0.25-0.81). Among the older group (≥2 years) (n = 24), the median age is 7.6 years (IQR 4.0-12.4).

### 3.2 Rater agreement for HU measurements

We measured the intra-rater agreement by calculating the intraclass correlation (ICC) on measurements on SL1 and SL2 in a randomly selected 10% scans, and ICCs were 0.97 and 0.95, respectively. The inter-rater agreement was previously reported for SL1 and SL2 with ICC 0.92 and 0.97, respectively(Ta, 2015).

### 3.3 Comparison between ROI and circular DOT methods

**Fig. 3:**
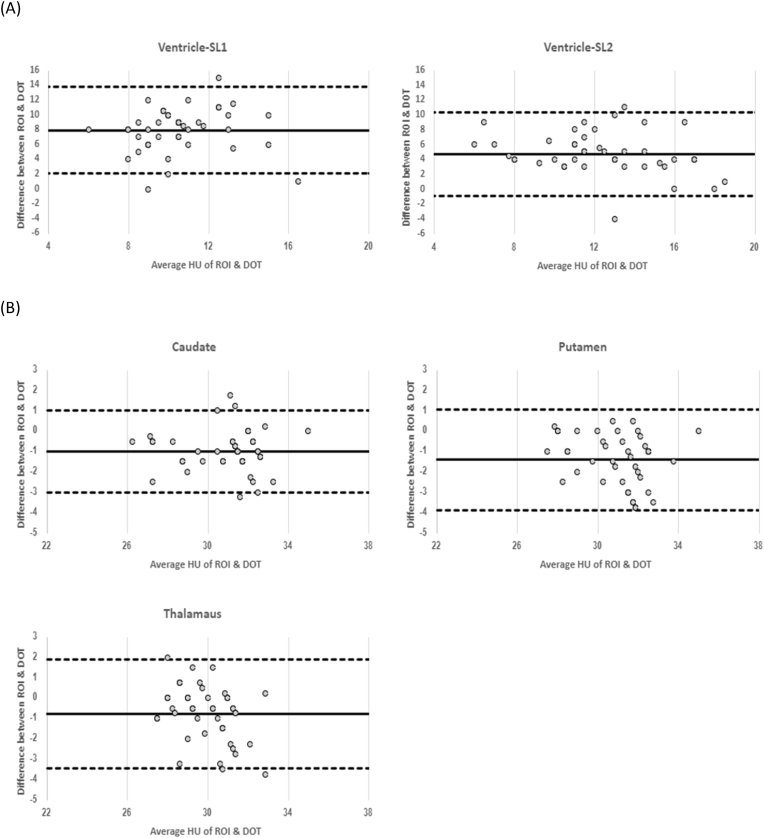
Bland-Altman plots show the mean HU difference of the ROI and DOT measurements in (A) ventricle slice 1 (SL1) and slice 2 (SL2); and (B) caudate nucleus, putamen, thalamus. The average ROI measurements were subtracted from the average of DOT measurements for each head CT (y-axis) plotted against the average HU measured for each head CT by both methods (x-axis). Bold lines represent the mean difference and dashed lines represent ± 1.96 SD.

HU measured with our segmented ROI and DOT methods in the CN, PT, and TM, and ventricles are shown in Table 2. In Fig. 3, the Bland-Altman plot indicates: 1) 95% limits-of-agreement (LoA) between the ROI and the DOT methods; 2) range of difference when subtracting DOT measurements from ROI measurements; and 3) overall mean difference (systematic bias). At CN: 1) LoA, −3.01 to 1.75 (SD = 1.04); 2) range between −3.25 and 1.75; and 3) bias of −0.98. At PT: 1) LoA, −3.88 to 1.09 (SD = 1.27); 2) range between −3.75 and 0.5; and 3) bias of −1.39. At TM: 1) LoA, −3.44 to 1.93 (SD = 1.37); 2) range between −3.75 and 2; and 3) bias of −0.76. At ventricle SL1: 1) LoA, 2.14 to 13.81 (SD = 2.98); 2) range between 0 and 15; and 3) bias of 7.98. At the ventricle SL2: 1) LoA, −0.78 to 10.47 (SD = 2.87); 2) range between −4 and 11; and 3) bias of 4.85.

**Table 2:** Median HU values in the four circular dot (DOT) areas and in the underlying regions of interest (ROI).

		All (n = 42)	
		Median	IQR
**ROI**	Ventricle SL1	15	12.3-16
	Ventricle SL2	15	12.3-17
**DOT**	Ventricle SL1	6	5.0-7.5
	Ventricle SL2	9	8-12
**ROI**	Caudate	31	29.3-32
	Putamen	30	29.3-31
	Thalamus	30	29-30.8
**DOT**	Caudate	31.9	30.1-32.7
	Putamen	32	30.5-33
	Thalamus	30.4	29-31.5

IQR, Interquartile range; SL1, slice 1; SL2, slice 2.

### 3.4 HU measurements and age

**Fig. 4:**
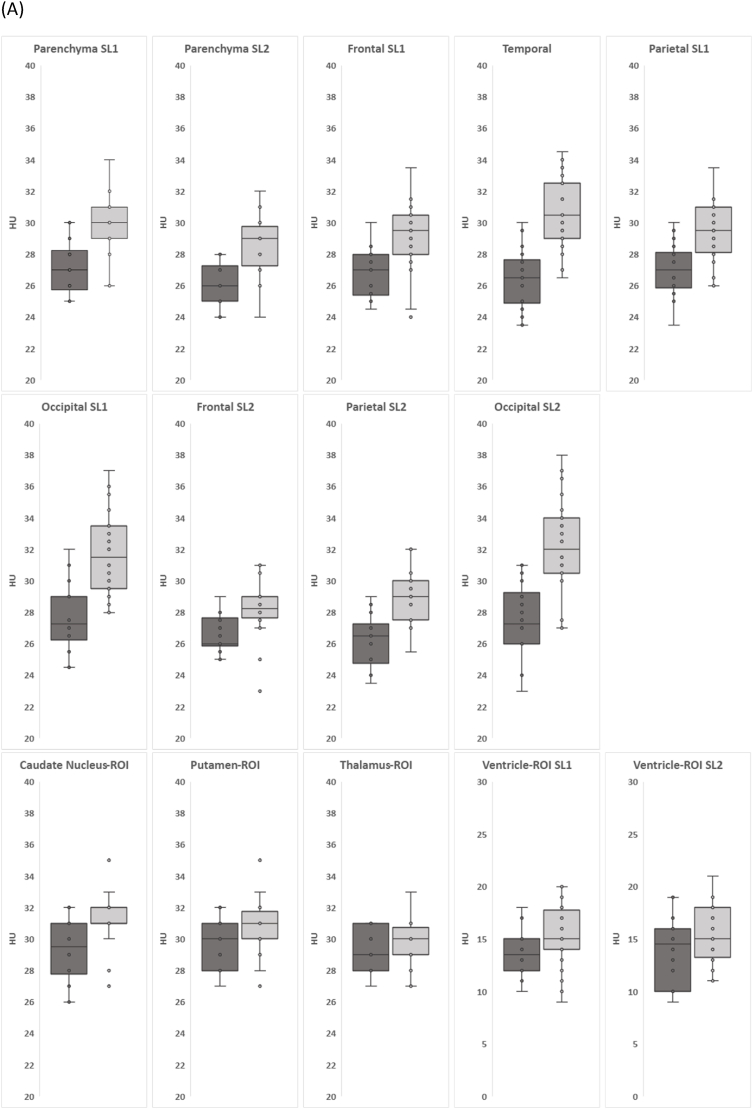
(A) Box and whisker plots show Hounsfield unit (HU, y-axis) from regions of interest (ROI) selected in slice 1 (SL1) and slice 2 (SL2), plotted by age group: dark gray box for less than 2 years old (n = 18) and light gray box for 2 years and older (n = 24). (B) Box and whisker plots show HU inside the circular dot (DOT) in gray matter and white matter in SL1 and SL2, deep nucleus (caudate, putamen, thalamus), and ventricles. Vertical axes of HU values range from 20 ≤ HU ≤ 40 for the ROI plots, from 18 ≤ HU ≤ 40 for gray matter and white matter DOT plots, and from 0 ≤ HU ≤ 30 for ventricles plots.

Median HU are reported in Table 3 and illustrated in Fig. 4 for each region's HU by age group with box plots (group <2 years in dark gray box (n = 18) and group ≥2 years in light gray box (n = 24).

**Table 3:** Age at scan and median Hounsfield unit (HU) in regions of interest (ROI) segmented and tissue compartments in circular dot (DOT).

Age group	All (n = 42)		0-2 y (n = 18)		2-18 y (n = 24)		
	Median	IQR	Median	IQR	Median	IQR	*p-value*
Age (in months)	33.5	6.5–98.8	5.5	3-9.75	91	48-148.5	
Age (in years)	2.8	0.54-8.23	0.46	0.25-0.81	7.6	4-12.38	
**HU-ROI**							
Parenchyma SL1			27	26-28	30	29-31	<0.005
Parenchyma SL2			26	25.3-27	29	27.5-29.3	<0.005
Frontal SL1			27	25.5-27.9	29.5	28-30.5	<0.005
Temporal			26.5	25-27.5	30.5	29-32.25	<0.005
Parietal SL1			27	26-28	29.5	28.4-31	<0.005
Occipital SL1			27.25	26.5-29	31.5	29.5-33.5	<0.005
Frontal SL2			26	26-27.4	28.25	27.9-29	0.0075∗
Parietal SL2			26.5	25-26.9	29	27.5-30	<0.005
Occipital SL2			27.25	26.3-28.9	32	30.5-34	<0.005
Caudate			29.5	28-31	31	31-32	0.041∗
Putamen			30	28-31	31	30-31.3	1∗
Thalamus			29	28.3-30.8	30	29-30.3	1∗
Ventricle SL1			13.5	12-15	15	14-17.3	1
Ventricle SL2			14.5	10.5-16	15	13.8-18	1

**HU-DOT**							
GM (Cortical) SL1			27.5	26-29	33	31-34	<0.005
GM (Cortical) SL2			28	26-29	32	31-34	<0.005
WM SL1			23.75	23-25.5	24	23-25	1∗
WM SL2			23	22-24	24	22.9-25	1
Caudate			30.3	28.8-31.7	32.5	31.7-33.3	0.016
Putamen			31	29.1-32.4	32.9	31.4-33.5	0.27
Thalamus			29.5	28.5-30.6	31.3	29.9-32.3	0.07
Ventricle SL1			6	5.3-7	6.5	5-8	1
Ventricle SL2			6	6.13-11	10	8-13.13	1

IQR, Interquartile range; y, years; SL1, slice 1; SL2, slice 2; GM, gray matter; WM, white matter; Adjusted p-value with Bonferroni correction for multiple comparisons from *t*-test for normally distributed data or Mann-Whitney *U* test (∗) for non-normally distributed data.

In general, HU is lower in the younger group compared to the older children. HU of parenchyma in SL1 and SL2 were significantly lower in the group <2 years than the group ≥2 years (26-27 vs. 29-30, p < 0.005). In all lobes, HU of group <2 years were lower than group ≥2 years (26-27.25 vs. 29-32, p < 0.005). Among the deep nucleus, only CN showed significantly lower HU in the group <2 years compared to the group ≥2 years (29 vs. 31, p = 0.041). Neither PT nor TM showed differences between age groups.

**Fig. 5:**
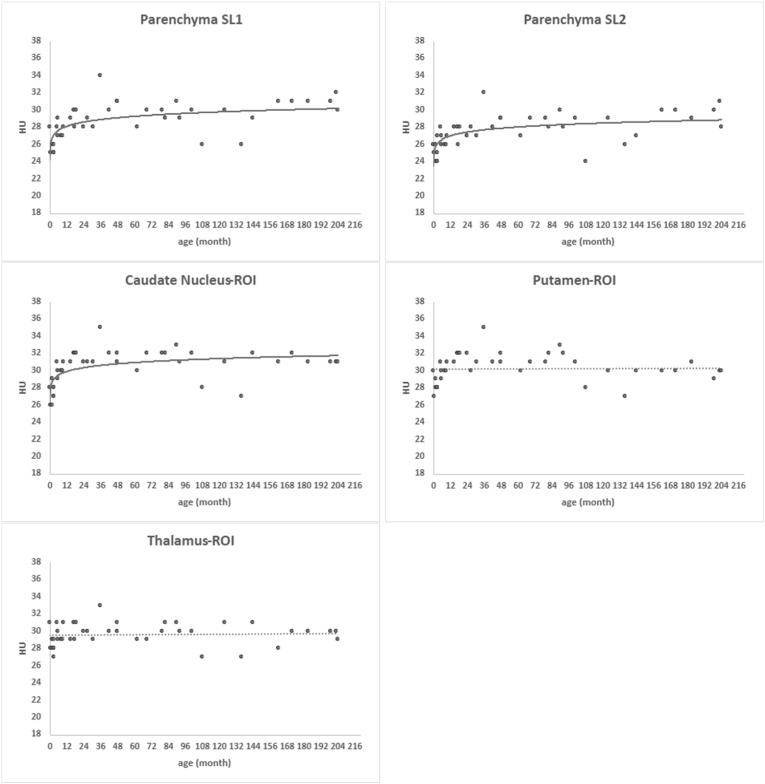
Scatterplots with fitted curve show HU in ROI as a function of age at the scan in parenchyma slice1 (SL1) and slice2 (SL2), CN, PT, TM, and GM (cortical) and WM from SL1 and SL2. Each individual curves' equations and R^2^ are as follows: 1) Parenchyma SL1: y = 26.424 x^∧^0.025, R^2^ = 0.3809; 2) Parenchyma SL2: y = 25.5 x^∧^0.0233, R^2^ = 0.3812; 3) CN: y = 28.394 x^∧^0.0211, R^2^ = 0.3386; 4) GM SL1: y = 26.843 x^∧^0.0403, R^2^ = 0.4875; and 5) GM SL2: y = 26.713 x^∧^0.039, R^2^ = 0.4941. PT, TM, WM SL1 and WM SL2 show close to flat linear lines with equations and R^2^ as follows: 1) PT: y = 0.0005x + 30.23, R^2^ = 0.0004; 2) TM: y = 0.0011x + 29.53, R^2^ = 0.0028; 3) WM SL1: y = −0.0031x + 24.26, R^2^ = 0.0145; and 4) WM SL2: y = 0.0061x + 23.086; R^2^ = 0.0423.

When plotting individual participants' HU on the y axis and age on the x axis and adding fitted curves (see Fig. 5), we observe that the median HU in parenchyma SL1 and SL2, CN, PT, and GM SL1 and SL2 increases with age in the younger group (<2 years). Median HU ranges from 26 to 28 and increases to 30 in parenchyma, to 32 in CN and PT, and to 34 in the cortical GM. We did not detect noticeable changes in HU with age in TM or in WM.

### 3.5 Gray-white matter ratio (GWR) formula differences

**Fig. 6:**
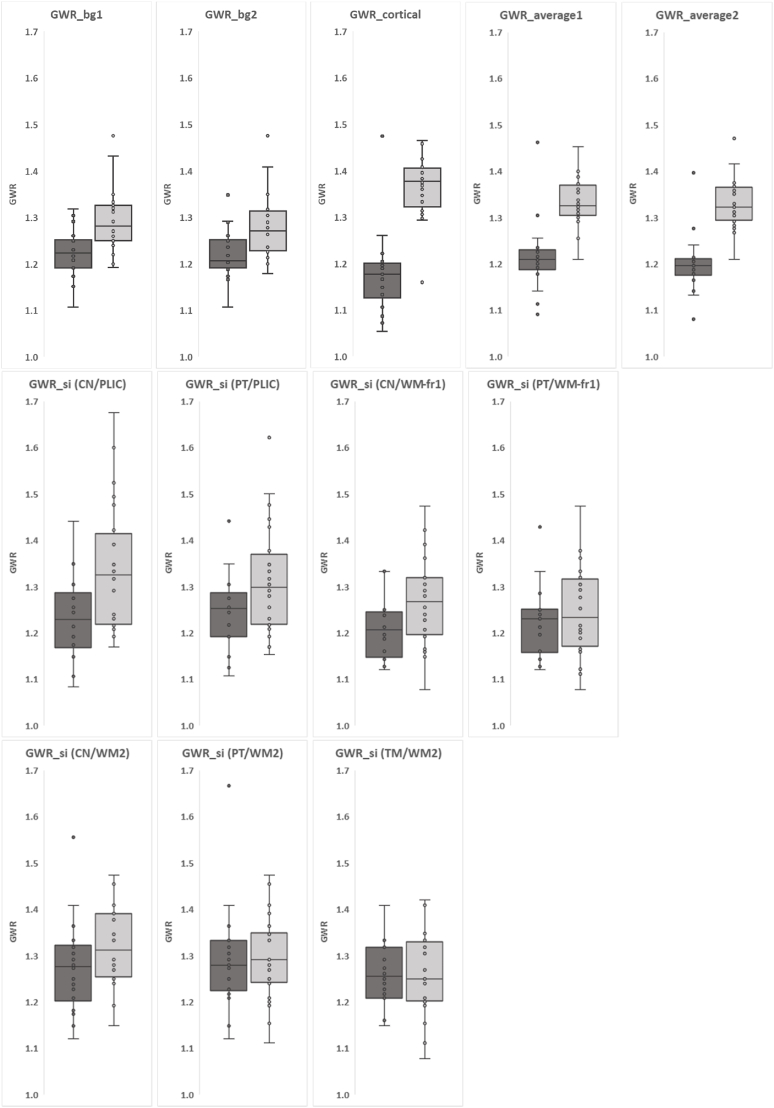
Box and whisker plots show median GWR calculated using 12 methods (see Table 1) on the samples. Vertical axes for GWR ≥1 as HU of gray matter is higher than white matter in normal brain. CN, caudate nucleus; PT, putamen; TM, thalamus, PLIC, posterior limb of internal capsule; WM, white matter; fr1, frontal lobe in slice 1; WM2, white matter in slice 2.

Median and IQR of GWR for each formula are reported in Table 4 and are illustrated with box plots in Fig. 6. The measured median GWR ranged from 1.18 to 1.28 in the group <2 years and between 1.23 and 1.38 in the group ≥2 years. GWR are lower in group <2 years than group ≥2 years in GWR_bg1 (1.22 vs. 1.28, p = 0.0073), GWR_cortical (1.18 vs. 1.38, p < 0.005), GWR_average1 (1.21 vs. 1.32, p < 0.005) and GWR_average2 (1.20 vs. 1.32, p < 0.005). The median values measured using GWR_simple approaches with CN (CN/PILC, CN/WM-fr1, CN/WM2), PT, or TM were not different between age groups.

**Table 4:** Gray-white matter ratio (GWR) median values and interquartile ranges.

Age group	0-2 y (n = 18)		2-18 y (n = 24)		
GWR	Median	IQR	Median	IQR	p-value
GWR_bg 1	1.22	1.19-1.25	1.28	1.25-1.33	0.0073
GWR_bg 2	1.21	1.19-1.25	1.27	1.23-1.31	0.04
GWR_cortical	1.18	1.13-1.20	1.38	1.33-1.40	<0.005
GWR_average 1	1.21	1.19-1.23	1.32	1.31-1.36	<0.005
GWR_average 2	1.20	1.18-1.21	1.32	1.30-1.36	<0.005
GWR_simple					
CN/PLIC	1.23	1.18-1.28	1.33	1.22-1.40	0.10
PT/PLIC	1.25	1.19-1.28	1.30	1.22-1.36	0.76
CN/WM-fr1	1.21	1.15-1.24	1.27	1.20-1.32	0.33
PT/WM-fr1	1.23	1.17-1.25	1.23	1.18-1.31	1
CN/WM2	1.28	1.21-1.31	1.31	1.26-1.39	0.97
PT/WM2	1.28	1.23-1.33	1.29	1.25-1.35	1
TM/WM2	1.26	1.21-1.31	1.25	1.21-1.32	1

For each GWR formula, refer to Table 1. GWR_bg1, calculated with basal ganglia (BG) without thalamus (TM); GWR_bg2, calculated with BG with TM; GWR_average1, all caudate nucleus (CN), putamen (PT), and cortical GM were added and divided by the total WM; GWR_average2, sum of GWR_bg1 and GWR_cortical divided by 2; GWR_simple (GWR_si) were calculated with one BG nucleus (CN, PT, or TM) divided with one of the white matter regions (PLIC, posterior limb of internal capsule; WM-fr1, white matter frontal slice 1; WM2, white matter slice 2); Adjusted p-value with Bonferroni correction for multiple comparison for the Mann-Whitney *U* test.

## 4 Discussion

In this study, we aimed to measure HU and GWR in head CT of pediatric normal control subjects to generate normative values for future use in studies of children with neurologic conditions. By reviewing our institution's clinical imaging archive, we assembled a cohort of scans from healthy individuals ranging in age from 1 month to 17 years. Children without neurologic conditions are very rarely scanned because of the risk associated with radiation from CT, which resulted in children less than 1 year old being overrepresented in the sample.

Using this cohort, we applied methods developed by our group to delineate regions for HU measurement on two axial slices. We report the instructions for using both our ROI and DOT methods. In studies requiring rapid estimation of HU, methods using circular dots are commonly used. Recently, some researchers have automated ROI selection using GM segmentation (Hanning et al., 2016; Kenda et al., 2021), or by computer-determined dot placement (Kenda et al., 2022). Pediatric studies are limited, but most report HU and GWR using dot methods (Lee et al., 2022; Starling et al., 2015; Yang et al., 2019a, Yang et al., 2019b), with one study using large circles (Tetsuhara et al., 2021). Two studies employed automated ROI for visually normal head CT scans (Cauley et al., 2018, 2021). Although ROI-based methods may provide more detailed information, they may be less accurate in regions of damaged brain tissue, whether applied manually or automatically. In contrast, methods with dots are easier and more consistent for HU measurement, which explains their widespread use. However, differences between dots and ROI methods remain rarely reported. To address this gap, although our ROI method requires more steps than the DOT method, both methods showed acceptable agreement, as indicated by the Bland-Altman plot. For all deep nuclei, bias between the two methods was small (approximately −1 HU), and over 90% of cases fell within the limits of agreement. We conclude that our ROI and DOT methods can estimate similar HU values with minimal bias for CN, PT, and TM.

We reviewed the literature to evaluate various approaches to segmentation and to GWR estimation in pediatric and adult populations. Many formulas for GWR exist, but no formula is considered a gold standard. We summarized the regions included in each calculation and contrasted them with our ROI and DOT methods. In future studies in target populations with hypoxic ischemic injury or traumatic brain injury, investigators may wish to compare the technical complexity, segmentation speed, reproducibility, and accuracy of different formulas.

A motivation for this study was to determine whether brain maturity or age affected GWR normative values. We found regional differences in HU and GWR on CT head imaging between normal control children younger than 2 years and older children. In this sample, the median HU values in CN, cortical GM, and brain parenchyma were lower in normal children younger than 2 years than in those older than 2 years. No differences were detected in PT, TM, and WM. We found that HU in brain parenchyma was as low as 24 at a month of life, increased through 3-5 years of age to 30, and plateaued after, as others have reported (Boris et al., 1987; Cauley et al., 2018). Few studies have studied age-related regional HU changes in children. One study showed increases in CN and cortical GM, to be most pronounced in the first years of life (Boris et al., 1987). Another study found that in the first year of life, HU in GM increased from 28 to 36, and from 23.7 to 26 in (Cauley et al., 2018). These differences are plausible, given that BG (CN, PT, and global pallidus) and TM differ developmentally. The BG develop from the telencephalon, in which medial global pallidus and lateral global pallidus and PT have different origins, while the TM develops from the diencephalon (Nunta-Aree et al., 2001; Torrico and Munakomi, 2023). The developmental differences in these regions may partly explain why the HU in CN showed age-related differences, whereas the TM did not; we can only speculate that we failed to detect differences in the PT because of the limited sample of controls. The number of subjects older than 2 years in our cohort of normal controls is a limitation of the study.

AHA guidelines for post-cardiac arrest care report that GWR<1.3 may be suggestive of cerebral edema in adults and that it may be a predictor of poor neurological outcome (Callaway et al., 2015). In adult cardiac arrest studies, some previously proposed GWR thresholds for predicting poor neurological outcome ranging from 1.1 to 1.24 with GWR_bg1/GWR_cortical/GWR_average2, from 1.09 to 1.22 with GWR_simple(CN/PLIC) and (CN/CC), from 1.06 to 1.21 with GWR_simple(PT/PLIC) and (PT/CC), and 1.13 with GWR_simple(TM/CC) (Choi et al., 2008; Na et al., 2018; Torbey et al., 2000).

Our results suggest adult cut-offs may not be suitable for young children, where normal controls less than 2 years demonstrated median GWR <1.3 (GWR_bg1, GWR_bg2, GWR_cortical, GWR_average1, GWR_average2, and GWR_simple, except for GWR_simple where GM was divided with WM-SL2). Similarly, others who studied cardiac arrest in children suggest that young children after cardiac arrest may have lower GWR compared to adults. Starling et al. proposed a GWR_bg1 threshold of 1.09 for predicting poor outcome in children <2 years old and 1.28 in ≥2 years (Starling et al.). Similarly, Yang et al. suggested a GWR_bg1 cutoff of 1.08 in less than 1 year and 1.15 in ≥1 year or 1.08 in age <4 years and 1.18 in ≥4 years (Yang et al., 2019a). They proposed thresholds for good neurologic outcome to be GWR_bg1 1.19, GWR_cortical 1.23, GWR_average2 1.21, GWR_simple(CN/PLIC) 1.23, GWR_simple(CN/CC) 1.18, GWR_simple(PT/PLIC) 1.22±, and GWR_simple(PT/CC) 1.14 (Yang et al., 2019a).

In a recently published study of adult patients with cardiac arrest (median age of 68 years), the predictive value of qualitative CT imaging assessment, manual, and automated GWR estimates was evaluated (Lang et al., 2024). A predefined GWR threshold <1.10, using either manual or automated methods, with 4 or 8 ROI in the basal ganglia, was found to be predictive of poor outcome (Lang et al., 2024). The same group reports that GWR <1.10 focused on ROI in the basal ganglia is very unlikely to be measured in the adult reference standard cohort studied (Lang et al., 2025).

When speculating on the sources of variation across different GWR estimates with normal pediatric controls, these may be influenced in part by age-related differences in HU in GM, but not in WM, which showed no age-related differences in our study. GWR calculated with CN (CN/PLIC, CN/WM-fr1, CN/WM2) is lower in the younger group than in the older group. In the other pediatric study that reported normal control values of GWR who were older (average 10.5 years (3.5-14.0) (Lee et al., 2022), they report a median GWR_bg1 of 1.27, GWR_cortical of 0.75, and GWR_average2 of 1.2. Our GWR_bg1 values are similar; in contrast, GWR_average2 in their cohort is lower, which may be explained by the very low values of their GM in their study.

Our data suggests that the age at image acquisition is essential to consider in children when using GWR in studies to predict outcomes, as some changes may be associated with normal brain maturation. HU and GWR in younger children are different from those in older children.

## 5 Limitations

Our study has intrinsic limitations. Healthy participant head CT scans are not approved for pediatric research, so we used clinically acquired images to generate the cohort of normal controls. Head CT imaging is rarely used in children without a neurologic complaint. Additionally, the radiation risk renders clinicians hesitant to use CT in patients if cranial US or brain MRI can be used as alternatives. Despite an extensive search of the imaging archives, we were unable to include many normal scans across the entire age range. Given the retrospective nature of scan selection, we could not confirm whether cases with no visible CT abnormalities at the time of acquisition subsequently developed neurological conditions a year after the index scan. Measurable changes on CT head in HU might not be sufficiently sensitive to exclude all neurological pathology. This study's head CTs were acquired using two models from the same manufacturer (using 100 or 120 kVp). Between manufacturer inter-scanner HU variability is reported; however, it is unclear whether significant differences exist within scanners of the same manufacturer (Oh et al., 2019). Cropp et al. showed that HU values were dependent on the scanner manufacturer and kilovoltage peak (kVp) (Cropp et al., 2013). As such, we suspect differences between scanner models of the same manufacturer used to generate this cohort may be minimal. Cauley et al. tested head CT scan in children aged between 0 and 2 years old using three different scanners and concluded that all three scans showed similar trends (Cauley, Hu, and Fielden 2021). Lastly, throughout the acquisition calendar years, scans were performed using age-adjusted dose-reducing protocols. These protocols may alter raw HU values; however, we speculate that the impact on GWR values was reduced.

## 6 Conclusion

Our study of pediatric head CT in normal controls suggests that HU measurements and GWR calculations should account for GM involvement and age, particularly in children younger than 2 years. Using our DOT method to analyze HU in two slices is feasible and shows reasonable agreement with our ROI method. Further studies should investigate which segmentation approaches enable the calculation of GWR that is most predictive of the severity of cerebral edema or hypoxic ischemic encephalopathy following cardiac arrest or traumatic brain injury. GWR on head CT may have a role in multimodal algorithms for the prognosis of hypoxic-ischemic encephalopathy; CT can be obtained in patients who cannot undergo MRI and isn't confounded by pharmacological sedatives or anesthetic agents, which can alter electroencephalography.

## CRediT authorship contribution statement

**Kie Honjo:** Writing – review & editing, Writing – original draft, Visualization, Validation, Software, Project administration, Methodology, Investigation, Formal analysis, Data curation. **Geraldine Goco:** Writing – review & editing, Resources, Project administration, Conceptualization. **Eva Ta:** Writing – review & editing, Software, Methodology, Conceptualization. **Suzanne Laughlin:** Writing – review & editing, Methodology, Investigation, Conceptualization. **Anne-Marie Guerguerian:** Writing – review & editing, Writing – original draft, Visualization, Validation, Supervision, Resources, Methodology, Investigation, Funding acquisition, Formal analysis, Data curation, Conceptualization.

## Funding

This research has been supported by a Garry Hurvitz Center for Brain and Mental Health, Health Outcomes award from The Hospital for Sick Children.

## Declaration of competing interest

The authors declare that they have no known competing financippaal interests or personal relationships that could have appeared to influence this study.

The original version of the Pediatric TBI CT Scan Tool's public disclosure's filling date was September 22, 2011.

## Data Availability

The authors do not have permission to share data.
