# Supplementary material for: Hounsfield unit and gray-white matter ratio estimates in pediatric normal controls used in studies evaluating head computed tomography
Source: Neuroimage Rep. 2026 Apr 30;6(2):100346. doi: 10.1016/j.ynirp.2026.100346 (PMC13146513; doi:10.1016/j.ynirp.2026.100346)
Supplement: Multimedia component 1 [file mmc1.docx]

**Table 5. Difference of HU between region of interest (ROI) and circular dot (DOT)**

| ​ | Mean difference​ | Difference in range​ | SD of difference​ | Limits of agreement​ |
| --- | --- | --- | --- | --- |
| Ventricle SL1​ | 7.98​ | 0 and 15​ | 2.98​ | 2.14, 13.81​ |
| Ventricle SL2​ | 4.85​ | -4 and 11​ | 2.87​ | -0.78, 10.47​ |
| Caudate​ | -0.98​ | -3.25 and 1.75​ | 1.04​ | -3.01, 1.06​ |
| Putamen​ | -1.39​ | -3.75 and 0.5​ | 1.27​ | -3.88, 1.09​ |
| Thalamus​ | -0.76​ | -3.75 and 2​ | 1.37​ | -3.44, 1.93​ |
